# Successful liver transplantation as rescue therapy in a patient with metastases from a vasoactive intestinal peptide producing neuroendocrine tumor

**DOI:** 10.1093/jscr/rjae371

**Published:** 2024-05-31

**Authors:** Mikkel Andreassen, Rajendra Singh Garbyal, Peter Nørgaard Larsen, Carsten Palnæs Hansen, Jens Hannibal, Peter Oturai, Ulrich Knigge, Nicolai Schultz

**Affiliations:** ENETS Center of Excellence, Copenhagen University Hospital, Rigshospitalet, 2100 Copenhagen, Denmark; Department of Endocrinology and Metabolism, Copenhagen University Hospital, Rigshospitalet, 2100 Copenhagen, Denmark; ENETS Center of Excellence, Copenhagen University Hospital, Rigshospitalet, 2100 Copenhagen, Denmark; Department of Pathology, Copenhagen University Hospital, Rigshospitalet, 2100 Copenhagen, Denmark; ENETS Center of Excellence, Copenhagen University Hospital, Rigshospitalet, 2100 Copenhagen, Denmark; Department of Surgery and Transplantation, Copenhagen University Hospital, Rigshospitalet, 2100 Copenhagen, Denmark; ENETS Center of Excellence, Copenhagen University Hospital, Rigshospitalet, 2100 Copenhagen, Denmark; Department of Surgery and Transplantation, Copenhagen University Hospital, Rigshospitalet, 2100 Copenhagen, Denmark; Department of Biochemistry, Copenhagen University Hospital, Bispebjerg, 2100 Copenhagen, Denmark; ENETS Center of Excellence, Copenhagen University Hospital, Rigshospitalet, 2100 Copenhagen, Denmark; Department of Clinical Physiology and Nuclear Medicine, Copenhagen University Hospital, Rigshospitalet, 2100 Copenhagen, Denmark; ENETS Center of Excellence, Copenhagen University Hospital, Rigshospitalet, 2100 Copenhagen, Denmark; Department of Endocrinology and Metabolism, Copenhagen University Hospital, Rigshospitalet, 2100 Copenhagen, Denmark; Department of Surgery and Transplantation, Copenhagen University Hospital, Rigshospitalet, 2100 Copenhagen, Denmark; ENETS Center of Excellence, Copenhagen University Hospital, Rigshospitalet, 2100 Copenhagen, Denmark; Department of Surgery and Transplantation, Copenhagen University Hospital, Rigshospitalet, 2100 Copenhagen, Denmark

**Keywords:** liver transplantation, neuroendocrine tumors, vasoactive intestinal peptide, VIPoma

## Abstract

This case report presents a 40-year-old patient with a vasoactive intestinal peptide (VIP) secreting high grade (Ki-67 39%) neuroendocrine tumor (NET) from the pancreas, for whom successful liver transplantation (LT) was carried out 8 years after resection of the primary tumor due to massive liver metastases. The transplantation was done as rescue therapy due to rapid progression and a devastating clinical condition requiring intravenous supplementation for 20 hours daily. The latest imaging carried out 18 months after transplantation is without signs of recurrence, and the patient is in good health with undetectable levels of VIP. According to the guidelines, LT is only recommended if Ki-67 is <20% and if there has been tumor control for more than 6 months prior to transplantation. Our case illustrates that LT is an option that should be considered for selected NET patients without extrahepatic involvement regardless of tumor grade and clinical condition.

## Introduction

Vasoactive intestinal peptide (VIP) secreting pancreatic neuroendocrine tumors (NET) (VIPomas), are extremely rare [1]. Patients typically present with watery diarrhea, hypokalemia, and achlorhydria (Verner Morrison syndrome, WDHA syndrome). Surgery remains the only curative treatment option. However, a considerable proportion of patients are diagnosed at advanced stages, where metastases, particularly to the liver, are common [1].

Liver transplantation (LT) is a viable treatment option for metastatic NET to the liver that has shown promising results in selected patients despite the complexities and challenges associated with the procedure. The transplantation offers a chance for complete removal of the tumor burden with improvement of hormonal symptoms, and a potential cure in cases refractory to other treatment modalities [2]. Nevertheless, careful patient selection is crucial, given the procedure’s inherent risks.

This case report presents a patient with a VIPoma, for whom successful LT was ultimately carried out 8 years after the initial diagnosis.

## Case presentation

In 2014 a 32-year-old female was referred due to a significant weight loss and severe diarrhea. A combined computed tomography (CT) ^64^Cu-DOTATATE positron emission tomography (PET) showed a NET in the head of the pancreas and elevation of plasma VIP. The patient underwent a pancreaticoduodenectomy (Whipple procedure) in June 2014. Pathologic examination revealed a VIPoma of 60 mm with a Ki-67 13% (G2) classified as pT3N0. After the operation all symptoms subsided, and VIP levels normalized.

The first relapse with two hepatic metastases was recorded in April 2015 and was treated with radiofrequency ablation (RFA). In February 2016, at least 10 additional hepatic metastases were identified. Chemotherapy with 5-fluorouracil and streptozocin was initiated, but due to tumor progression treatment was swiftly substituted by four cycles of Peptide Receptor Radionuclide Therapy (PRRT) with ^177^Lu-Dotatate with the last cycle administered in April 2017.

In June 2019, the patient had progressive disease and renewed liver RFA and two additional PRRT cycles was performed. One year later, the patient had recurrence of severe diarrhea and progression of liver metastases that were treated with surgical resection and concomitant RFA in February 2021. The patient had

temporary symptom relief, but due to rapid disease progression, extensive palliative liver surgery with debulking was performed in September 2021. Pathologic examination now revealed a transformation of tumor cells to G3 NET with a Ki-67 index of 39%. An attempt to control symptoms with somatostatin analog therapy was made in summer 2021 but had to be discontinued due to side effects.

In December 2021, the patient was hospitalized due to severe metabolic acidosis, hypokalemia, more than 10 episodes of daily diarrhea and further weight loss. Despite reintroduction of octreotide along with prednisolone, there was no discernible improvement. The patient’s condition demanded increasing intravenous supplementation for 20 hours daily, as she had about 3 liters of watery diarrhea/day. Her typical daily regimen included the following: 400-mmol oral potassium, 10.000-mg oral bicarbonate, 200-mmol intravenous potassium, 100-mL intravenous bicarbonate 8.4% every 4 hours, 1.5-L intravenous sodium chloride, 1.5-L parenteral nutrition, 34-mmol intravenous phosphate in 500-mL isotonic saline and 300-μg subcutaneous octreotide three times daily.

**Figure 1:**
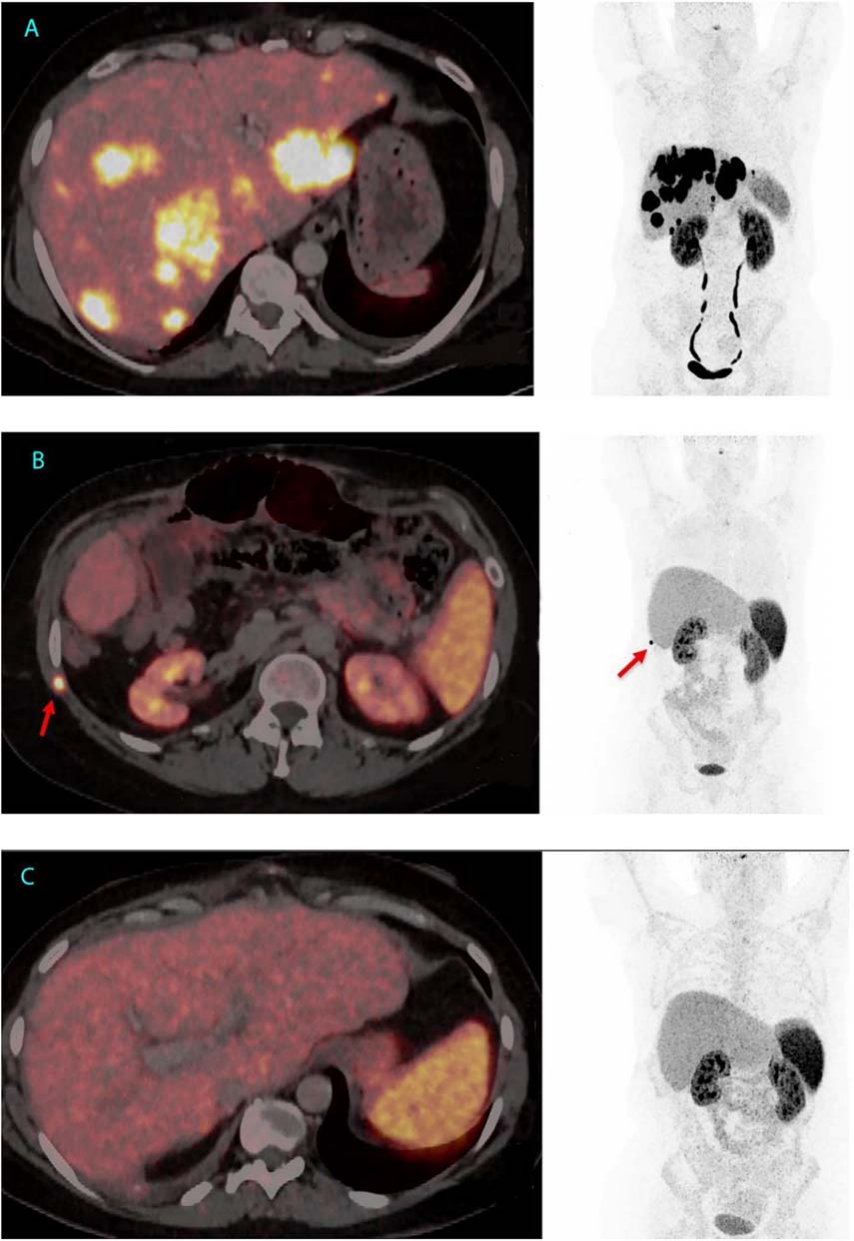
^64^Cu-DOTATATE PET/CT from 2 June 2022, 7 weeks before LT with extensive hepatic involvement but no extrahepatic metastases (A). ^64^Cu-DOTATATE PET/CT 15 months after LT revealed an implantation metastasis in the thoracic wall (B). Latest ^64^Cu-DOTATATE PET/CT performed 18 months after LT and 3 months after resection of the thoracic metastasis showed no signs of recurrence (C).

Given her declining health, the patient was placed on the LT waitlist in March 2022. After a 7-month hospital stay, she underwent an LT in June 2022. Figure 1 presents the final ^64^Cu-DOTATATE PET/CT prior to transplantation, demonstrating extensive hepatic involvement without extrahepatic metastases. The LT was a technical demanding and high-risk procedure because of the previous Whipple procedure, liver resections and complicated biliary leakages. Abdominal wall was closed POD 2 with a biological mesh. The transplantation was complicated by necrotizing pancreatitis, successfully treated by endoscopic, transgastric necrosectomy.

After LT, plasma VIP normalized, but the patient was hospitalized for an additional 4 months for rehabilitation and treatment of different infections. In November 2023, 15 months after LT a routine ^64^Cu-DOTATATE PET/CT revealed a potential implantation metastasis in the thoracic wall where a drain had previous been placed. Surgery was conducted and pathological examination confirmed the presence of a NET metastasis with a Ki-67 index of 10%.

**Figure 2:**
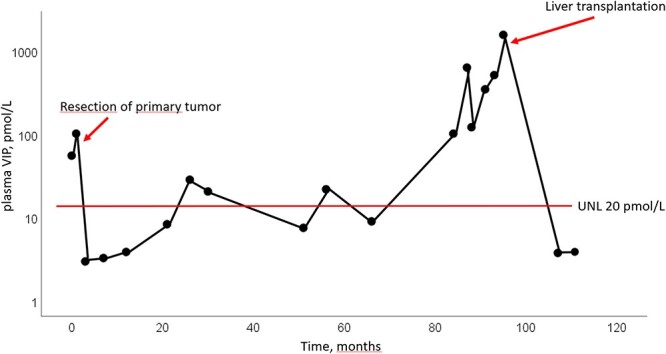
Changes in plasma levels of VIP. Latest measurement from January 2024 was <3.8 pmol/L. UNL = upper normal level.

In the most recent ^64^Cu-DOTATATE PET/CT scan from January 2024, conducted 18 months after LT, there was no signs of recurrence. Currently, the patient is in good health, has started part-time work and has no hormone-related symptoms, since LT serum VIP has been below level of detection (<3.8 pmol/L) (Figs 1 and 2).

## Discussion

We describe an until now successful outcome of LT in a 40-year-old woman diagnosed with VIPoma 8 years prior to transplantation, after all medical and surgical treatment options seemed exhausted. To our knowledge, only eight prior case reports of LT for VIPoma have been published. Five of these in a review from 2011 which concluded that the prognosis following LT for VIPoma appeared to be more favorable compared to other NET. Notably, all five patients were reported to be alive at post-transplantation intervals ranging from 12 to 123 months [3]. Age at transplantation ranged from 41 to 62 years. Another case report is from 2010 describing a 9-year-old who underwent LT and was still alive 25 years later. In the last two case reports, no details on outcome after transplantation were given.

Current guidelines for LT in patients with NET can be found in the Milan Criteria from 2016, and in the United Network for Organ Sharing criteria. According to the guidelines, LT is only recommended if the Ki-67 is <20% and if there has been tumor control for more than 6 months prior to transplantation. Our patient progressed to G3 with a Ki-67 of 39% in the latest biopsies before surgery, and there was significant progression up to the transplantation. We acknowledge that LT was a high-risk procedure with a significant risk of a fatal outcome, but the case illustrates that LT is an option that should be considered for selected NET patients without extrahepatic involvement regardless of tumor grade and clinical condition. Furthermore, it is noteworthy that the outcome after LT for VIPoma seems to be better than that otherwise observed in metastases from pancreatic NET.

## References

[1] Hofland J , Falconi M, Christ E, et al. European Neuroendocrine Tumor Society 2023 guidance paper for functioning pancreatic neuroendocrine tumour syndromes. J Neuroendocrinol 2023;35:e13318. 10.1111/jne.13318. Available from. http://www.ncbi.nlm.nih.gov/pubmed/37578384. 37578384

[2] de Souza M Fernandes E , Garcia Kyt CV, de Mello FPT, et al. Liver transplantation in gastroenteropancreatic neuroendocrine tumors. Front Oncologia 2022;12:1001163. 10.3389/fonc.2022.1001163. Available from. http://www.ncbi.nlm.nih.gov/pubmed/36844922. PMC9947829 36844922

[3] Máthé Z , Tagkalos E, Paul A, et al. Liver transplantation for hepatic metastases of neuroendocrine pancreatic tumors: a survival-based analysis. Transplantation 2011;91:575–82. 10.1097/TP.0b013e3182081312. Available from. http://www.ncbi.nlm.nih.gov/pubmed/21200365. 21200365

